# Perception of Internalized Stigma in Parents of Children With Autism Spectrum Disorder: Investigating the Effects of Depression, Anxiety, and Family Functioning

**DOI:** 10.7759/cureus.73860

**Published:** 2024-11-17

**Authors:** Fatma Efe, Huseyin Aksoy, Fesih Ok, Elif Kocak, Serkan Gunes

**Affiliations:** 1 Radiology, Cukurova University Faculty of Medicine, Adana, TUR; 2 General Practice, University Hospital Southampton, Southampton, GBR; 3 Urology, Adana City Training and Research Hospital, Adana, TUR; 4 Child Psychiatry, Adana City Training and Research Hospital, Adana, TUR

**Keywords:** anxiety, autism spectrum disorder (asd), depression, family med, stigma

## Abstract

Introduction

Autism spectrum disorder (ASD) is a pervasive neurodevelopmental disorder that impacts communication, behavior, and social interaction. It is well-documented that parents of children with ASD often experience psychiatric conditions like anxiety and depression. Nevertheless, research on the perception of internalized stigma, family functioning, and their interconnections is scarce. The main objective of our study was to analyze the internalized stigma perceptions, depression, anxiety, and family functioning among parents or caregivers of children diagnosed with ASD.

Methods

This study was conducted on 102 cases and 101 healthy controls in a cross-sectional case-control study involving patients aged 3-12 years. The research was carried out at the Child Psychiatry Clinic of Adana City Training and Research Hospital and three Family Health Centers in Adana from October 15 to December 15, 2022. Sociodemographic data, family history, families' knowledge levels about autism, and information about their social lives were collected using a face-to-face structured questionnaire. The Internalized Stigma of Mental Illness (ISMI), Hospital Anxiety and Depression (HAD), and Family APGAR scales were administered.

Results

The mean ISMI score of the case group was 67.31±8.91. According to the HAD Anxiety scale score, 60.8% of the case group and 14.9% of the control group were at high risk (p<0.001). According to the HAD Depression Scale, 52% of the case group participants and 25.7% of the control group were at high risk (p<0.001). The rate of severe familial dysfunction was 18.6% in the case group and 3.0% in the control group (p<0.001).

Conclusion

Stigma, anxiety, depression, and family functioning in parents of children diagnosed with ASD lead to various adverse outcomes. Further studies are necessary to prevent these negative experiences for families of children with ASD and to facilitate the integration of these children into society.

## Introduction

Autism spectrum disorder (ASD) is a neurodevelopmental disorder that can manifest in the early stages of life with social and communication disabilities, limited interactions, limited interests, and repetitive behaviors [1,2]. The prevalence of ASD has been determined as 1 in 59 in Turkey, and its frequency is increasing daily. This makes ASD a significant public health issue. Developmental delays such as social communication deficiencies, repetitive behaviors, and motor development deficiencies are frequently observed in ASD [2]. Mothers whose children have developmental delays such as speech delay, mental retardation, physical disability, and autism can experience high levels of depression and stress [3]. A mother's depression can cause emotional and cognitive problems in children by hindering her ability to behave consistently and responsibly over time. Therefore, early diagnosis and proper treatment of parents with depression are crucial for enhancing the health of both the child and the parents [4].

Children with ASD can display very variable patterns of language pathologies and may exhibit aggressive behavioral problems. These individuals also have limitations in expressing themselves, which may lead to societal stigmatization [5]. When people interact with someone with autism, they may feel different and strange. Caregivers who notice this may be affected psychosocially and feel stigmatized [6]. Stigma, such as avoidance and discrimination, manifests as a sense of shame and the expectation of prejudice from others, hindering individuals from discussing their experiences and seeking help. Internalized stigma refers to the internalization of negative attitudes and beliefs towards people with mental illness, significantly impacting people's self-perception and general well-being [7]. On the other hand, family functioning is defined as the supportive relationships between parents and family members [8]. Domains considered fundamental in family functionality include adaptability, partnership, growth, affection, love, and decision-making [9]. Illness and incapacity are shared experiences and represent one of the biggest challenges for families because the psychosocial problems created by a dependent person affect the family system as a whole [10]. Ongoing stress and burden (OSB) can profoundly impact family functioning, including the roles and responsibilities that parents take on [11].

In the literature, numerous studies focus on anxiety and depression in parents of children with ASD. However, fewer studies explore internalized perceptions of stigma and family functioning. The primary objective of our study was to investigate the internalized perceptions of stigma, depression, anxiety, and family functioning among caregivers of children diagnosed with ASD. Our secondary objective was to examine the relationship between the internalized perceptions of stigma, depression, anxiety, and family functioning.

## Materials and methods

Sample

The present study was conducted between October 15 and December 15, 2022. As a case group, caregivers of patients between the ages of 3 and 12 who were diagnosed with autism and applied to the clinic at Adana City Training and Research Hospital Child Psychiatry Clinic were included. Parents of healthy children between the ages of 3 and 12 who applied to three Family Health Centers in Adana province were listed as the control group. Parents of children with similar age and sociodemographic characteristics were recruited.

The inclusion criteria for the patient group were as follows: 1) age between 3 to 12 years, 2) a diagnosis of ASD according to DSM-5 criteria, 3) caregivers not having intellectual disabilities or COVID-19 infection, 4) willingness to participate in the study and signing the consent form. The inclusion criteria for the control group were: 1) age between 3 to 12 years, 2) not having a chronic physical illness or psychiatric disorder according to DSM-5, 3) caregivers not having intellectual disabilities or COVID-19 infection, 4) willingness to participate in the study and signing the consent form.

On the other hand, the exclusion criteria for the patient group were as follows: 1) age younger than 3 years or older than 12 years, 2) being an adopted child, 3) caregivers having intellectual disability or COVID-19 infection, 4) not volunteering to participate in the study or sign the consent form. The exclusion criteria for the control group were the same as those for the patient group.

**Figure 1:**
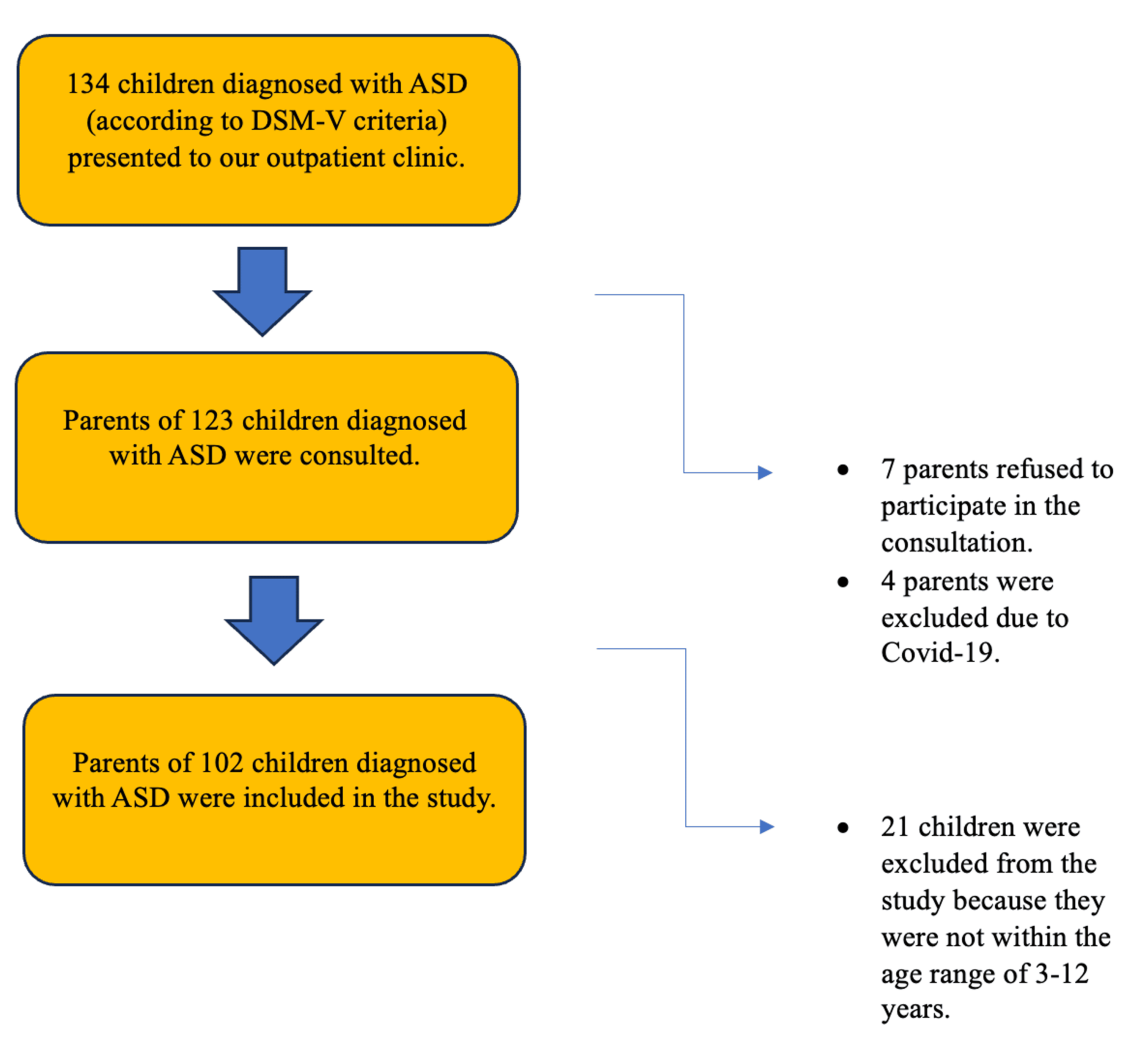
Case group flow diagram.

**Figure 2:**
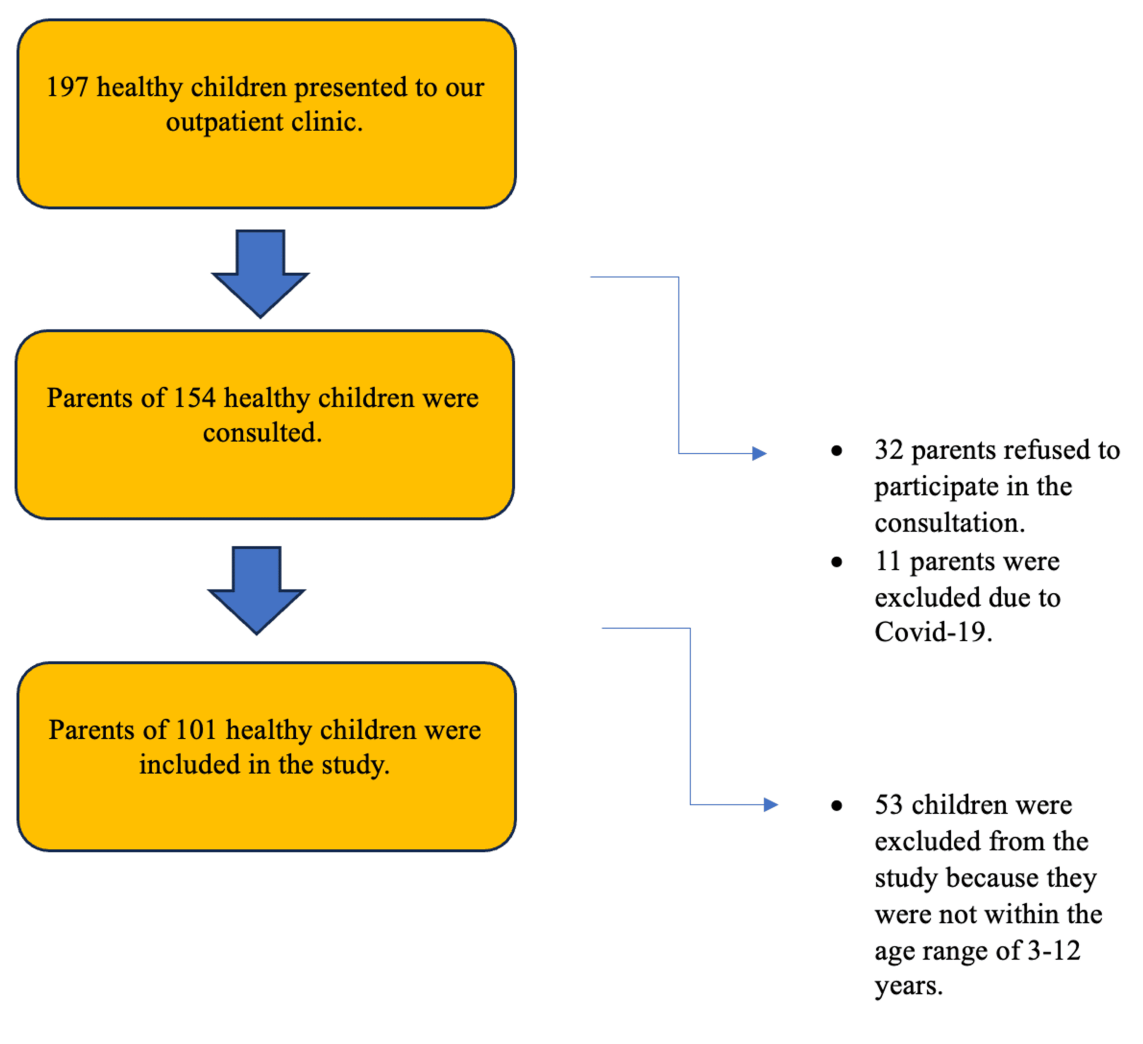
Control group flow diagram.

A total of 203 volunteers, 102 in the case group and 101 in the control group, were included in the study (Figures 1-2). The caregivers of the children participating in the study were verbally informed about the study's purpose and the interview duration. They were also provided with a written informed consent form, which was given to all participants, ensuring that only volunteers took part in the study. The study received approval from the Ethics Committee through a decision made by the Clinical Research Ethics Committee of Adana City Training and Research Hospital on 06.10.22, with the reference number 2174.

Sample size

A priori power calculation analysis was conducted. For the power calculation, a previous study examining the Family APGAR total score in children with ASD and controls was taken as a reference [12]. In the mentioned study, the mean family APGAR scores of the ASD and control groups were 15.17 (SD: 0.907) and 16.60 (SD: 3.688), respectively. In the present study, it was estimated that at least 55 subjects in each group would be required to detect an effect size (0.53) between the ASD and control groups at the 80% power level with a type 1 error of 5%.

Scales

The structured questionnaire administered to the parents and caregivers in the case and control groups included sociodemographic data forms (patient sociodemographic data, family history, levels of family knowledge about autism, inquiries about their social lives), the Hospital Anxiety Depression Scale, and Family APGAR scale. The Internalized Stigmatization Scale in Mental Illnesses (ISMI) was administered only to the parents and caregivers in the case group.

Internalized Stigma of Mental Illness (ISMI) scale

This is a 29-item self-report scale that evaluates internalized stigma, developed by Boyd-Ritsher et al. (2003). The scale comprises five subscales: "alienation" (1, 5, 8, 16, 17, 21), "approval of stereotypes" (26, 10, 18, 19, 23, 29), "perceived discrimination" (3, 15, 22, 25, 28), "social withdrawal" (4, 9, 11, 12, 13, 20), and "resistance to stigmatization" (7, 14, 24, 26, 27). The items on the scale are answered on a four-point Likert scale ranging from "strongly disagree" (1 point) to "strongly agree" (4 points). The items of the stigma resistance subscale (items; 7, 14, 24, 26, 27) are calculated inversely. The total score of the ISMI varies between 4 and 116, and there is no cut-off score. High scores indicate a higher level of internalized stigma. The Turkish validity and reliability of the scale were established by Ersoy and Varan (2007) [13].

Hospital Anxiety and Depression (HAD) scale

This scale, used to determine the risk level of anxiety and depression in individuals applying to primary health care services, was developed by Zigmond and Sinaith. The Turkish validity and reliability study was conducted by Aydemir et al. [14]. It is a self-assessment scale consisting of 14 questions. Odd-numbered items measure the risk of anxiety, while even-numbered items measure the risk of depression. The responses are scored on a four-point Likert scale. For anxiety questions (1, 3, 5, 6, 8, 10, 11, and 13), scoring decreases in severity from 3 to 0; for depression questions (2, 4, 7, 9, 12, and 14), scores increase from 0 to 3. The maximum score for each subscale is 21, with cut-off values of 10 for anxiety and 7 for depression in the Turkish form, which were used in this study.

Family APGAR scale

Many tools are used to measure family functions. Many valid methods can be excessively long. Clinicians may choose a shorter assessment as part of their routine consultation with patients or during screening. One of the tools that provides this brief assessment is Family APGAR, developed by Smilkstein. The validity and reliability study of the scale was conducted by Özcan et al. in 2011 [15] This scale, which enables people to evaluate their perceptions about the functionality of their families with 5 closed-ended questions, helps to understand the weaknesses in family functioning and to focus on the problems in this regard. This scale is a screening test that allows physicians to quickly gain insight into family function. Family functionality is listed in 5 parameters; adaptation, partnership, growth, affection, and decision-making. The word APGAR comes from the initials of each of these parameters. For each proposition on the scale, there are three different options that indicate the frequency of satisfaction. These are sorted into 0 (almost never), 1 (sometimes), and 2 (almost always). By adding the scores obtained from each parameter in the scale, a total score of 21 is obtained. The range of points that can be obtained varies between 0 and 10. A high score is an indicator of family functionality satisfaction.

Statistical analysis

Data was analyzed using the statistical software SPSS 26.0. Descriptive statistics were conducted on the sociodemographic data of the participants, family history, families' knowledge levels about autism, data on questions about their social lives, and the scores from the scales used in the study. Descriptive statistical methods such as mean, standard deviation, median, frequency, ratio, minimum, and maximum were utilized. The normal distribution was evaluated using Skewness and Kurtosis tests. Student's t-test was employed for normally distributed parameters, while the Mann-Whitney U test was used for non-normally distributed parameters. The one-way ANOVA test was utilized for comparisons among more than two groups with normal distribution, and the Kruskal-Wallis test was used for those without normal distribution. The chi-square test was used for categorical data involving two or more groups. Spearman's Correlation Analysis was employed to evaluate relationships between numerical data. A significance level of p<0.05 was considered significant.

## Results

A total of 203 people participated in our study. Approximately 50% (n=102) of the participants were in the case group, and 50% (n=101) were in the control group. In the case group, 86.3% (n=88) were male, and 13.7% (n=14) were female. In the control group, 51.5% (n=52) were male, and 48.5% (n=49) were female.

The mothers' average age in the case group was 35.29±6.97 years, while it was 36.65±6.27 years in the control group. The fathers' average age was 39.84±7.06 years in the case group, and 39.47±7.24 years in the control group. Upon evaluating the age variables, there was no statistically significant difference between the groups (Table 1).

**Table 1:** Sociodemographic data. *The Chi-square test was used for statistical analysis. **The independent samples t-test was used for statistical analysis.

Sociodemographic characteristics (n=203)		Case group	Control group	P-value
		n=102	n=101	
		%	%	
Gender (child)	Male	88 (86.3%)	52 (51.4%)	<0.001*
	Female	14 (13.7%)	49 (48.6%)	
Living place	Province	83 (81.3%)	80 (80%)	0.394*
	County	14 (13.7%)	18 (18.00%)	
	Village	5 (4.9%)	2 (2.00%)	
Mother's employment status	Homemaker	75 (73.5%)	38 (37.6%)	<0.001*
	Running	17 (16.7%)	57 (56.4%)	
	Left the job	10 (9.8%)	6 (6.0%)	
Marital status of parents	Married	93 (91.2%)	89 (89%)	0.022*
	Single	0 (0.00%)	5 (5.00%)	
	Reserved	9 (8.8%)	6 (6%)	
Mother's educational background	Illiterate	4 (3.9%)	1 (1%)	<0.001*
	Primary school	26 (25.5%)	8 (7.9%)	
	Secondary school	16 (15.7%)	6 (5.9%)	
	High school	29 (28.4%)	19 (18.8%)	
	University	27 (26.5%)	67 (66.3%)	
Father's educational background	Primary school	21 (20.6%)	4 (4%)	<0.001*
	Secondary school	19 (18.6%)	5 (5%)	
	High school	34 (33.3%)	22 (21.8%)	
	University	28 (27.5%)	70 (69.2%)	
	Case group	Control group	t-value	P-value
	(Avg.±ss)	(Avg.±ss)		
Father’s age (years)	39.84±7.06	39.47±7.24	0.376	0.707**
Mother's age (years)	35.29±6.97	36.65±6.27	-1.46	0.146**

The mean ISMI score for men was 67.06±9.03, and for women, it was 68.93±8.22 (Table 2).

**Table 2:** Descriptive data of the ISMI in case group participants. ISMI: Internalized Stigma of Mental Illness.

ISMI	Mean±SD	Min-Max
	67.31±8.91	51-88

According to the Family APGAR scale scoring, the case group had severe dysfunction in 18.6% (n=19), moderate dysfunction in 40.2% (n=41), and high functionality in 41.2% (n=42). In contrast, the control group had severe dysfunction in 3% (n=3), moderate dysfunction in 27.7% (n=28), and high functionality in 69.3% (n=70). A statistically significant difference was observed between the groups (p<0.001), with a higher rate of severe dysfunction in the case group.

According to the HAD scale for anxiety scoring, 39.2% (n=40) of the case group were at low risk, while 60.8% (n=62) were at high risk. In contrast, 85.1% (n=86) of the control group were at low risk, and 14.9% (n=15) were at high risk. A statistically significant difference was observed between the groups (p<0.001). The HAD Anxiety Scale indicated a higher rate of participants at high risk for anxiety in the case group.

According to the HAD scale for depression scoring, 48% (n=49) of the case group were at low risk, and 52% (n=53) were at high risk. In contrast, 74.3% (n=75) of the control group were at low risk, while 25.7% (n=26) were at high risk. A statistically significant difference was found between the groups (p<0.001). The proportion of participants at high risk for depression was higher in the case group (Table 3).

**Table 3:** Table on the comparison of the scores of the scales used in the study according to the groups. *The Chi-square test was used for statistical analysis. APGAR: Appearance, Pulse, Grimace, Activity, and Respiration; HAD: Hospital Anxiety and Depression.

		Case group	Control group	X	P-value
Family APGAR scale	Severe dysfunction	19 (18.6%)	3 (3%)	21.081	<0.001*
	Moderately dysfunctional	41 (40.2%)	28 (27.7%)		
	Highly functional	42 (41.2%)	70 (69.3%)		
HAD Anxiety	Low risk	40 (39.2%)	86 (85.1%)	45.486	<0.001*
	High risk	62 (60.8%)	15 (14.9%)		
HAD Depression	Low risk	49 (48%)	75 (74.3%)	14.675	<0.001*
	High risk	53 (52.00%)	26 (25.7%)		

There was a moderate negative correlation between the parents' time spent visiting friends/relatives and the ISMI score (r = -0.229, p = 0.021).

There was a moderate negative correlation between parental self-time and HAD Anxiety (r = -0.267, p < 0.001), and between the parents' time spent visiting friends/relatives and HAD Anxiety (r = -0.324, p < 0.001).

There was a moderate negative correlation between parental self-time and HAD Depression (r = -0.251, p < 0.001), and similarly between the parents' time spent visiting friends/relatives and HAD Depression (r = -0.241, p < 0.001). On the other hand, a moderate positive correlation was found between the parents' self-time and the Family APGAR scale (r = 0.214, p = 0.002). Additionally, a moderate positive correlation was observed between the parents' time spent visiting friends/relatives and the Family APGAR scale (r = 0.269, p < 0.001) (Table 4).

**Table 4:** Correlation table of the relationships between the scales used in the study and selected parental data. r: correlation coefficient, P: p-value Spearman correlation analysis was used. APGAR: Appearance, Pulse, Grimace, Activity, and Respiration; HAD: Hospital Anxiety and Depression; ISMI: Internalized Stigma of Mental Illness; ASD: Autism spectrum disorder.

		ISMI	HAD Anxiety	HAD Depression	Family APGAR
Mother's educational background	r	-0.135	-0.176	-0.153	0.137
	P	0.176	0.012	0.029	0.052
Father's educational background	r	-0.036	-0.218	-0.129	-0.07
	P	0.719	0.002	0.067	0.32
Family income	r	-0.244	-0.224	-0.137	0.172
	P	0.014	0.001	0.051	0.014
Mom/Dad taking time for themself	r	-0.02	-0.267	-0.251	0.269
	P	0.842	<0.001	<0.001	<0.001
Mom/Dad's contact with friends	r	-0.229	-0.324	-0.241	0.214
	P	0.021	<0.001	0.001	0.002
Getting training on ASD	r	-0.232	-0.262	-0.259	-0.039
	P	0.019	0.008	0.009	0.695

In the correlation analysis of scale scores, a moderate positive correlation was found between ISMI scores and HAD Anxiety (r=0.555, p<0.001), and between ISMI scores and HAD Depression (r=0.584, p<0.001). Additionally, a moderate negative correlation was found between ISMI scores and the Family APGAR scale (r=-0.269, p=0.006).

A high-severity positive correlation was found between HAD Anxiety and HAD Depression (r=0.844, p<0.001). Additionally, a moderate negative correlation was found between HAD Anxiety and the Family APGAR scale (r=-0.348, p<0.001).

A moderate negative correlation was found between HAD Depression and the Family APGAR scale (r = -0.333, p < 0.001) (Table 5).

**Table 5:** Correlation table of the relationships between the scales used in the study. r: correlation coefficient; P: p-value. Spearman correlation analysis was used. APGAR: Appearance, Pulse, Grimace, Activity, and Respiration; HAD: Hospital Anxiety and Depression; ISMI: Internalized Stigma of Mental Illness; ASD: Autism spectrum disorder.

		ISMI	HAD Anxiety	HAD Depression	Family APGAR
ISMI	r	-	0.555	0.584	-0.269
	P	-	<0.001	<0.001	0.006
HAD Anxiety	r	0.555	-	0.844	-0.348
	P	<0.001	-	<0.001	<0.001
HAD Depression	r	0.584	0.844	-	-0.333
	P	<0.001	<0.001	-	<0.001
Family APGAR	r	-0.269	-0.348	-0.333	-
	P	0.006	<0.001	<0.001	-

## Discussion

The present study investigated the relationship between internalized stigma perception, depression, anxiety, and family functioning of parents diagnosed with ASD. These symptoms were found to be moderately related to each other. Additionally, we examined the relationship between education, family income, parental socialization status, internalized stigma, anxiety, depression, and family functioning of parents with children who have ASD.

In some studies, stigma has been found to decrease with increased parental education level [16]. According to a review by Liao X et al., stigmatization is less common among caregiver parents who receive education about autism [17]. Hsiao CY et al. found that a low level of education was associated with decreased family functioning in people diagnosed with schizophrenia and their caregivers [18]. An intervention study on families with children with attention deficit and hyperactivity disorder reported that family functionality increased in families that received psychoeducational support [19]. Having information about the condition of individuals or families can reduce their stress and future anxiety, thereby improving their situation in terms of depression, anxiety, stigma, and family functioning. For this reason, information sessions and training, especially in family health centers that are easily accessible to families, can be organized.

According to Ran MS et al., a strong relationship was found between decreased family income and increased stigmatization in some psychiatric disorders [20]. Yıldız M et al. revealed that family income and economic problems increase the caregiver burden for those diagnosed with ASD and schizophrenia, thereby increasing internalized stigma and depression in caregivers [21]. Economic difficulties can affect individual relationships within the family and the care of children [22]. A study on families with chronically ill children showed that family income affects family adaptation, child health, and family functionality [23]. To reduce the effects of economic hardship on families regarding stigma, anxiety, and family dysfunction, psychological and financial support should be provided to family caregivers.

According to Lee LC et al., families of children with ASD were more likely to leave their jobs and participate less in social activities [24]. Alibekova R et al. reported that parents without the support of friends and lacking social acceptance experience higher levels of stress, anxiety, and depression [25]. Organizing various activities and training can help families participate in social activities and receive social support. Kousha M et al.'s study in Iran with 127 mothers of children diagnosed with ASD indicated that these mothers had a higher risk of depression and anxiety [16]. Almansour et al.'s retrospective study highlighted a high risk of depression and anxiety in families of children with ASD [26]. Öz B et al.'s study on mothers with 69 children diagnosed with ASD revealed high levels of stress, anxiety, and depression in these mothers [4]. Benson PR and Karlof KL study on 217 parents of children with ASD showed that the level of anxiety experienced by parents is directly related to symptoms of depression, with an increase in anxiety leading to an increase in depression symptoms [27]. Pisula E's article reported a high risk of stress and depression among parents of children with ASD [28]. Lyu QY et al.'s study on 180 parents of children with ASD found low family functioning among these parents [29]. Lei X et al.'s study on families with 167 children with ASD indicated low family functionality among parents of children with ASD [30]. Jackson SZ et al.'s study on 101 mothers of children with ASD in a double control group revealed higher depression scores in mothers of children with ASD and lower family functionality [31].

In the literature, numerous studies explain the relationship between internalized stigma and various types of stigmatization with depression and anxiety. According to a study conducted by Chan KK and Leung DC, it was found that internalized or indirect stigmatization of individuals is directly and positively related to the anxiety and depression of parents caring for children diagnosed with ASD [32]. Another study by Benson PR reported that parents of children with ASD experienced significantly higher levels of anger, anxiety, and depressive behaviors, and the stress they faced was linked to depression [33]. Alshahrani MS and Algashmari H's study suggested that stigma experienced by parents of children with autism could mediate their stressful and depressive states, with a high prevalence of depression among parents of children with ASD [34]. In a study by Khusaifan SJ and El Keshky ME involving 93 parents of children with ASD in Saudi Arabia, it was revealed that over half of the participants exhibited symptoms of anxiety and depression. The study also highlighted the importance of family support and marital satisfaction in mitigating the negative impacts of autism-related conditions on parents' psychological well-being [35]. Additionally, a review by Gardiner E and Iarocci G indicated that family functioning deteriorates when parents face high stress due to their children's behavioral issues and developmental delays [36].

An increase in stigma among parents of children diagnosed with ASD has various negative consequences, such as increasing the risk of anxiety and depression and reducing family functioning. Determining the social and internalized causes of stigmatization is a precaution to minimize stigmatization in primary care and hospitals, and, if possible, to prevent it. Training the community and affected individuals to avoid stigmatization may be essential. Primary care family physicians play an important role because the diagnosis of ASD is increasing day by day. It shows symptoms in early childhood, a medical condition that can be addressed with the necessary guidance in early childhood. Family physicians must inform families about the developmental delays they notice in early childhood and guide them without delay.

Limitations of the study

Firstly, the case and control groups differed in terms of their family income and educational status. Secondly, the family APGAR scale we used to assess family functioning was administered only to the participants' nuclear families, excluding the extended family. Thirdly, there was no clinical assessment of the participants for anxiety and depression in the study; the responses were solely self-reported.

## Conclusions

Parents of children with ASD had moderately high stigma scores and a significantly higher risk of anxiety and depression. The rate of severely dysfunctional families was notably higher among parents of children with ASD. As the level of education and involvement in social life related to ASD increased for these parents, stigmatization, anxiety, and depression decreased significantly. With improved family functionality, the engagement of parents of children with ASD in social activities also increased. However, as stigma intensifies within these families, anxiety and depression levels also rise, resulting in a notable decline in family functioning.

An increase in stigma among parents of children diagnosed with ASD has various negative consequences, such as increasing the risk of anxiety and depression and reducing family functioning. Identifying the social and internalized causes of stigmatization is a preventive measure to minimize stigma in primary care and hospitals and, if possible, to prevent it. It is essential to train the community and affected individuals to prevent stigmatization.

Primary care family physicians play an essential role as the diagnosis of ASD is increasing daily. ASD manifests symptoms in early childhood, a medical condition that can be managed with appropriate guidance. Family physicians must inform families about the developmental delays observed in early childhood and provide timely guidance.

## References

[1] GÖLBAŞI H, Demirel Y, Karaca SN, Çiçek AU, SARI SA (2021). Prevalence of autism spectrum disorder (ASD) in Sivas Provincial Center and ASD awareness of health workers in family health centers. Çukurova Med J.

[2] Sanchack KE, Thomas CA (2016). Autism spectrum disorder: primary care principles. Am Fam Physician.

[3] Park GA, Lee ON (2022). The moderating effect of social support on parental stress and depression in mothers of children with disabilities. Occup Ther Int.

[4] Öz B, Yüksel T, Nasiroğlu S (2020). Depression-anxiety symptoms and stigma perception in mothers of children with autism spectrum disorder. Noro Psikiyatr Ars.

[5] Pyszkowska A, Rożnawski K, Farny Z (2021). Self-stigma and cognitive fusion in parents of children with autism spectrum disorder. The moderating role of self-compassion. PeerJ.

[6] Kheir NM, Ghoneim OM, Sandridge AL, Hayder SA, Al-Ismail MS, Al-Rawi F (2012). Concerns and considerations among caregivers of a child with autism in Qatar. BMC Res Notes.

[7] Tilahun D, Hanlon C, Fekadu A, Tekola B, Baheretibeb Y, Hoekstra RA (2016). Stigma, explanatory models and unmet needs of caregivers of children with developmental disorders in a low-income African country: a cross-sectional facility-based survey. BMC Health Serv Res.

[8] Johnson N, Frenn M, Feetham S, Simpson P (2011). Autism spectrum disorder: parenting stress, family functioning and health-related quality of life. Fam Syst Health.

[9] de Oliveira SC, Pavarini SC, Orlandi Fde S, de Mendiondo MS (2014). Family functionality: a study of Brazilian institutionalized elderly individuals. Arch Gerontol Geriatr.

[10] Rodríguez-Sánchez E, Pérez-Peñaranda A, Losada-Baltar A, Pérez-Arechaederra D, Gómez-Marcos MÁ, Patino-Alonso MC, García-Ortiz L (2011). Relationships between quality of life and family function in caregiver. BMC Fam Pract.

[11] DePape AM, Lindsay S (2015). Parents' experiences of caring for a child with autism spectrum disorder. Qual Health Res.

[12] Serrano L, Vela E, Martín L (2023). Analysis of the functioning of families of children with autism spectrum disorder: a psychometric study of the family APGAR scale. Int J Environ Res Public Health.

[13] Ersoy MA, Varan A (2007). [Reliability and validity of the Turkish version of the internalized stigma of mental illness scale]. Turk Psikiyatri Derg.

[14] Aydemir O, Güvenir T, Küey L, Kültür S (1997). Validity and reliability of Turkish version of hospital anxiety and depression scale. Turkish J Psych.

[15] Ozcan S, Duyen V, İncecik Y (2011). Use of family APGAR scale in family medicine: study of Turkish. J Turkish Fam Phys.

[16] Kousha M, Attar HA, Shoar Z (2016). Anxiety, depression, and quality of life in Iranian mothers of children with autism spectrum disorder. J Child Health Care.

[17] Liao X, Lei X, Li Y (2019). Stigma among parents of children with autism: a literature review. Asian J Psychiatr.

[18] Hsiao CY, Lu HL, Tsai YF (2020). Factors associated with family functioning among people with a diagnosis of schizophrenia and primary family caregivers. J Psychiatr Ment Health Nurs.

[19] Öztürk FÖ, Ekinci M (2023). The effect of psycho-education given to mothers of children with attention deficit hyperactivity disorder on mother-child interaction and family functionality. J Child Adolesc Psychiatr Nurs.

[20] Ran MS, Zhang TM, Wong IY (2018). Internalized stigma in people with severe mental illness in rural China. Int J Soc Psychiatry.

[21] Yıldız M, Demir Y, Kırcalı A, İncedere A (2021). Caregiver burden in schizophrenia and autism spectrum disorders: a comparative study. Psychiatry Investig.

[22] Lugo-Gil J, Tamis-LeMonda CS (2008). Family resources and parenting quality: links to children's cognitive development across the first 3 years. Child Dev.

[23] Zhang Y, Wei M, Shen N, Zhang Y (2015). Identifying factors related to family management during the coping process of families with childhood chronic conditions: a multi-site study. J Pediatr Nurs.

[24] Lee LC, Harrington RA, Louie BB, Newschaffer CJ (2008). Children with autism: quality of life and parental concerns. J Autism Dev Disord.

[25] Alibekova R, Kai Chan C, Crape B (2022). Stress, anxiety and depression in parents of children with autism spectrum disorders in Kazakhstan: prevalence and associated factors. Glob Ment Health (Camb).

[26] Miniarikova E, Vernhet C, Peries M, Loubersac J, Picot MC, Munir K, Baghdadli A (2022). Anxiety and depression in parents of children with autism spectrum disorder during the first COVID-19 lockdown: Report from the ELENA cohort. J Psychiatr Res.

[27] Benson PR, Karlof KL (2009). Anger, stress proliferation, and depressed mood among parents of children with ASD: a longitudinal replication. J Autism Dev Disord.

[28] Pisula E (2002). [Parents of children with autism: recent research findings]. Psychiatr.

[29] Lyu QY, Yu XX, Wang JL, Wang XY, Ke QQ, Liu D, Yang QH (2022). Self-esteem and family functioning mediates the association of symptom severity and parental affiliate stigma among families with children with ASD. J Pediatr Nurs.

[30] Lei X, Kantor J (2021). Social support and family functioning in Chinese families of children with autism spectrum disorder. Int J Environ Res Public Health.

[31] Jackson SZ, Pinto-Martin JA, Deatrick JA, Boyd R, Souders MC (2024). High depressive symptoms, low family functioning, and low self-efficacy in mothers of children with autism spectrum disorder compared to two control groups. J Am Psychiatr Nurses Assoc.

[32] Chan KK, Leung DC (2021). Linking child autism to parental depression and anxiety: the mediating roles of enacted and felt stigma. J Autism Dev Disord.

[33] Benson PR (2006). The impact of child symptom severity on depressed mood among parents of children with ASD: the mediating role of stress proliferation. J Autism Dev Disord.

[34] Alshahrani MS, Algashmari H (2021). The moderating effect of financial stress and autism severity on development of depression among parents and caregivers of Autistic children in Taif, Saudi Arabia. J Family Med Prim Care.

[35] Khusaifan SJ, El Keshky ME (2021). Social support as a protective factor for the well-being of parents of children with autism in Saudi Arabia. J Pediatr Nurs.

[36] Gardiner E, Iarocci G (2012). Unhappy (and happy) in their own way: a developmental psychopathology perspective on quality of life for families living with developmental disability with and without autism. Res Dev Disabil.

